# easytrack: A napari plugin for automated parameter tuning in cell tracking

**DOI:** 10.17912/micropub.biology.002090

**Published:** 2026-05-26

**Authors:** Tim Huygelen, Alan Lowe, Yanlan Mao, Pablo Vicente-Munuera

**Affiliations:** 1 Laboratory for Molecular Cell Biology, University College London, London, UK; 2 Institute for the Physics of Living Systems, University College London, London, UK; 3 Chan Zuckerberg Initiative, Redwood City, CA, US

## Abstract

Life is in constant movement, even microscopic bodies like cells. To understand cellular dynamics, or how cells move and interact, we analyse time-lapse microscopy images that highlight cellular structures like membranes and nuclei. The bioimage community has developed automated tracking algorithms to follow cells over time and space, but these tools often require extensive manual parameter tuning and technical expertise. *easytrack* democratises cell tracking by providing an intuitive graphical interface and automating the parameter optimisation process, making these powerful algorithms accessible to researchers without computational backgrounds.

**Figure 1. Napari-easytrack workflow and results:** **A** ) Workflow of the napari plugin easytrack. The parameter tuning widget (top) uses ground truth annotations to optimise btrack parameters via Bayesian optimisation, using Optuna and traccuracy for the optimisation and evaluation process respectively. The tracking widget (bottom) applies these optimised parameters to new datasets. **B-C** ) Tracking Accuracy normalisation of the AOGM (TRA) and ( **C** ) Acyclic oriented graphs matching measure (AOGM) scores for the diﬀerent datasets: ”DIC-C2DH- HeLa”, ”Fluo-N2DH-GOWT1”, ”PhC-C2DH-U373”, and ”wing disc”. Each dataset contains two sets of images. Results are average between the set of images in each dataset. Yellow bar: btrack default cell parameters. Blue bar: easytrack default epithelial cells preset. Red bar: predictions from the trained algorithm with the other set of images it was not trained with (n=10). Green bar: tracking results using the same set of images easytrack was trained on (n=10). Purple bar: random parameters that create valid tracking images (n=10). Note that for TRA higher is better. In contrast, for AOGM lower is better.

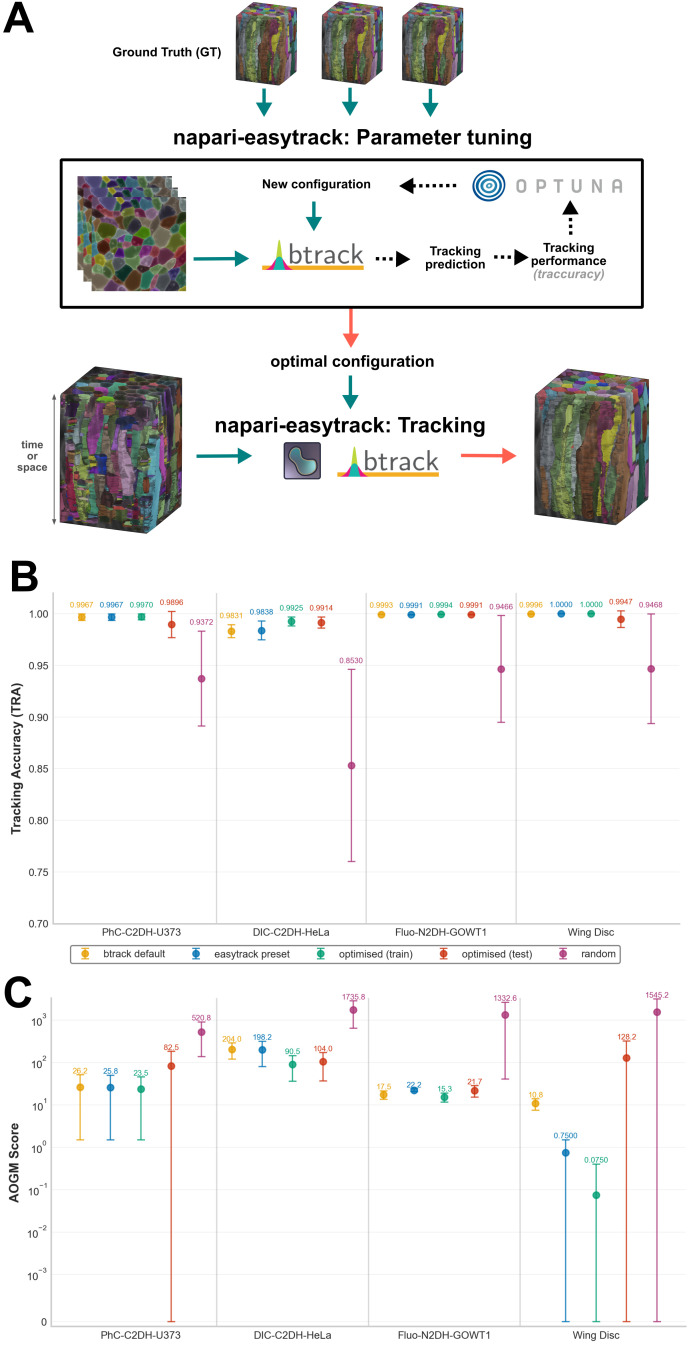



## Description

Cell tracking, the process of following individual cells through time and space, is essential for quantifying dynamic cellular behaviours such as migration, division, and morphological changes. These measurements underpin research into tissue healing (Tetley, 2019), developmental biology (Valon, 2021), and cancer progression (Hong, 2016). Despite advances in computational methods (Ulman, 2017, and Maska, 2023), many tracking algorithms require careful tuning of multiple parameters to achieve accurate results (Loffler 2021, and Chenouard, 2014). Current cell tracking software falls into two categories: general-use tools with modest accuracy, or high-performance algorithms like btrack (Bove, 2017, and Ulicna, 2021) that require extensive manual parameter tuning. This manual tuning is labour-intensive, requires expertise, and often needs repetition for diﬀerent datasets or experimental conditions. For instance, for btrack, a Bayesian cell tracking algorithm, this involves configuring 18 parameters across its motion and hypothesis models. In contrast, while deep learning approaches to cell tracking have shown impressive results (Soelistyo, 2023; Sugawara et al., 2025; Lange et al., 2025), classical algorithms (Bove, 2017, and Ulicna, 2021) retain important advantages: they require no training data for the tracker itself, and their parameters are directly interpretable as physical properties of the cells and images (e.g. expected migration speed, division rate, search radius).

The lack of accessible parameter tuning tools creates a barrier between technological capability and practical application. Researchers often resort to default parameters or limited manual exploration of the parameter space, potentially missing optimal configurations for their specific datasets. While hyperparameter optimisation frameworks like Optuna (Akiba, 2019) have proven eﬀective in machine learning contexts, their application to cell tracking software remains limited, with no existing tools providing an accessible interface for biologists.

easytrack addresses this gap by implementing automated parameter tuning within the napari ecosystem (https://zenodo.org/record/3555620), leveraging Bayesian optimisation techniques including Tree-structured Parzen Estimators (TPE) (Ozaki, 2022). By automating what was previously a manual, iterative process, easytrack makes high-accuracy cell tracking accessible to researchers without computational expertise whilst reducing the time required from hours to minutes. Sophisticated cell tracking algorithms often remain underutilised due to their complexity (Soelystio, 2023). *easytrack* addresses this usability barrier by automating parameter tuning, enabling:

1. Reproducible research: Parameter configurations can be saved, shared, and reused, reducing variability across studies.

2. Eﬃciency gains: Optimisation completes in tens of minutes compared to hours or days of manual tuning.

3. Accessibility: Non-experts can leverage state-of-the-art tracking without deep algorithmic knowledge.

To test easytrack’s eﬃcacy, we benchmarked it versus btrack’s default parameters for cells using diﬀerent datasets. To do that, we used some of the non-epithelial datasets from the Cell Tracking Challenge (CTC) (Ulman, 2017, and Maska, 2023) and two epithelial datasets (’wing disc’) from a 2D+time wound healing dataset (Tetley, 2019) and a 3D one from (Paci, 2026; https://www.ebi.ac.uk/biostudies/BioImages/studies/S-BIAD843). First, we predicted the trajectories of the cells in our datasets using btrack’s default set of parameters for cells ( Figure 1B- C). We compared these results with our default easytrack config preset for epithelial cells ( Figure 1A ). To faithfully compare the results from diﬀerent tracking algorithms, we used two of the metrics the CTC uses: Acyclic oriented graphs matching measure (AOGM score), defined such that a score of 0 represents a perfect result; and Tracking Accuracy normalisation of the AOGM (TRA), whose perfect score would be represented as 1 and 0 as the worst. Overall, we obtained very similar results for all the datasets with btrack and easytrack ( Figure 1B- C, yellow and blue bar). However, easytrack can train a dataset-specific model in about 30 minutes to obtain a better fit, whereas doing so manually would increase the tuning time substantially. We trained 10 times with each set of images, and obtained that AOGM and TRA scores were significantly improved after using the learned parameters on the same dataset ( Figure 1B- C, ’train’). Furthermore, to test each model generalisability, we predicted the tracks of the cells using the parameters learned from the other set of images within the same dataset. Indeed, results worsened in some datasets compared to ‘train’, but overall improved upon default parameter settings, suggesting its generalisability within the same type of images. Finally, to reliably assess whether easytrack can find optimal parameters, we used random parameters that created valid tracking images and results (see Methods). Compared to random valid parameters, all the different set of parameters perform better (Fig. B-C, 'random'). To sum up, we have shown easytrack’s versatility by tracking different types of cells and its improvements over the default set of parameters.

Advances in artificial intelligence have produced powerful segmentation tools for microscopy images (Stringer, 2021). However, for complex 3D segmentations, a tracking step is required to get an accurate result (Paci and Vicente-Munuera, 2025). easytrack has already been integrated into napari-EpiTools (https://github.com/epitools/epitools), a tool for analysing packed epithelial tissues. Previously, napari-EpiTools lacked a tracking algorithm with a simplified graphical user interface. This integration allows EpiTools’ user base to benefit from automated parameter tuning for accurate 3D tracking. While btrack is one of the most popular and robust plugins in napari, it is not specialised for packed epithelial tissues. Given that easytrack can be trained to track any kind of cells in a relatively short amount of time, it fills a gap in the napari community for easy-to-use tracking tools for both epithelial tissues and non-epithelial cells. Furthermore, its implementation as a standalone plugin ensures broader accessibility across the napari community.

## Methods

easytrack is implemented as napari-easytrack, a plugin for the napari multi-dimensional image viewer, integrating seamlessly with the growing napari bioimage analysis ecosystem. The design philosophy emphasises composability over reinvention, building upon established open-source tools: btrack for tracking (Ulicna, 2021, and Bove, 2017), Optuna for optimisation (Akiba, 2019), and traccuracy for evaluation (https://github.com/live-image-tracking-tools/traccuracy). The plugin provides two complementary widgets (Figure 1):

- Parameter tuning widget: Automates the process of finding optimal btrack parameters for given data using Bayesian optimisation. Optuna (Akiba, 2019) is a hyperparameter optimisation framework that can be used to to find the best solution that minimises (or maximises) a function. Users provide ground truth annotations (cells that have been segmented and tracked), and easytrack's widget optimises btrack’s 18 parameters by minimising the diﬀerence between predicted and ground truth tracking. The optimisation proceeds in two phases: first, 64 random trials sample parameters uniformly to explore the search space; then 128 trials guided by a Tree-structured Parzen Estimator (TPE) preferentially sample from parameter regions that produced good tracking scores. In this way, all parameter tuning start with random configurations and no prior knowledge. TPE efficiently navigates btrack’s 18-dimensional parameter space by modelling the distributions of parameters that fall above or below a performance threshold, avoiding the exponential scaling of grid search. Each trial runs the full tracking and evaluation pipeline, minimising the AOGM score from the Cell Tracking Challenge (Maska, 2014). Optimised parameters are saved as JSON configuration files for subsequent use.

-Tracking widget: Applies previously optimised or manually created parameter configurations to new segmented time-lapse or 3D images. The widget parses JSON configuration files, executes btrack with the specified parameters, and displays tracked cells with their trajectories overlaid for visual inspection and manual correction if needed. Both widgets include segmentation preprocessing functionality to remove small artefacts that can compromise tracking accuracy.

We used some of the Cell Tracking Challenge datasets to compare easytrack with btrack and random parameters, both trained and untrained. Given that Cell Tracking Challenge ground-truth annotations do not always cover every cell in every frame (the gold truth (GT) segmentation may annotate only a subset of cells, while silver truth (ST) provides denser but noisier coverage), we took two measures to ensure a fair evaluation. First, we merged GT and ST segmentation masks on a per-pixel basis, with GT taking priority, and relabeled every cell using its tracking (TRA) identity to maintain temporal consistency. Second, we filtered the ground-truth tracking graph (man_track.txt) to retain only those cell lineages whose segmentation masks were actually present in the input provided to the tracker, preventing spurious false-negative penalties for cells the tracker never had the opportunity to detect. TRA was computed via the traccuracy library using the CTC matching protocol. In addition, we relied on CTC matching to compute the different evaluation metrics TRA and AOGM. Thus, there are some parameters that would result in incorrectly tracked images, which would lead to an error in the traccuracy library's CTC matching protocol. For Figure 1B –C ('Random'), we discarded the ones that gave an error and kept only those with valid tracking scores.

Code is accessible via: https://github.com/timsmsmsm/easytrack .

## References

[R1] Akiba Takuya, Sano Shotaro, Yanase Toshihiko, Ohta Takeru, Koyama Masanori (2019). Optuna. Proceedings of the 25th ACM SIGKDD International Conference on Knowledge Discovery & Data Mining.

[R2] Bove Anna, Gradeci Daniel, Fujita Yasuyuki, Banerjee Shiladitya, Charras Guillaume, Lowe Alan R. (2017). Local cellular neighborhood controls proliferation in cell competition. Molecular Biology of the Cell.

[R3] Chenouard N, Smal I, de Chaumont F, Maška M, Sbalzarini IF, Gong Y, Cardinale J, Carthel C, Coraluppi S, Winter M, Cohen AR, Godinez WJ, Rohr K, Kalaidzidis Y, Liang L, Duncan J, Shen H, Xu Y, Magnusson KE, Jaldén J, Blau HM, Paul-Gilloteaux P, Roudot P, Kervrann C, Waharte F, Tinevez JY, Shorte SL, Willemse J, Celler K, van Wezel GP, Dan HW, Tsai YS, Ortiz de Solórzano C, Olivo-Marin JC, Meijering E (2014). Objective comparison of particle tracking methods.. Nat Methods.

[R4] Hong Y, Fang F, Zhang Q (2016). Circulating tumor cell clusters: What we know and what we expect (Review).. Int J Oncol.

[R5] Löffler K, Scherr T, Mikut R (2021). A graph-based cell tracking algorithm with few manually tunable parameters and automated segmentation error correction.. PLoS One.

[R6] Maška M, Ulman V, Delgado-Rodriguez P, Gómez-de-Mariscal E, Nečasová T, Guerrero Peña FA, Ren TI, Meyerowitz EM, Scherr T, Löffler K, Mikut R, Guo T, Wang Y, Allebach JP, Bao R, Al-Shakarji NM, Rahmon G, Toubal IE, Palaniappan K, Lux F, Matula P, Sugawara K, Magnusson KEG, Aho L, Cohen AR, Arbelle A, Ben-Haim T, Raviv TR, Isensee F, Jäger PF, Maier-Hein KH, Zhu Y, Ederra C, Urbiola A, Meijering E, Cunha A, Muñoz-Barrutia A, Kozubek M, Ortiz-de-Solórzano C (2023). The Cell Tracking Challenge: 10 years of objective benchmarking.. Nat Methods.

[R7] Maška M, Ulman V, Svoboda D, Matula P, Matula P, Ederra C, Urbiola A, España T, Venkatesan S, Balak DM, Karas P, Bolcková T, Streitová M, Carthel C, Coraluppi S, Harder N, Rohr K, Magnusson KE, Jaldén J, Blau HM, Dzyubachyk O, Křížek P, Hagen GM, Pastor-Escuredo D, Jimenez-Carretero D, Ledesma-Carbayo MJ, Muñoz-Barrutia A, Meijering E, Kozubek M, Ortiz-de-Solorzano C (2014). A benchmark for comparison of cell tracking algorithms.. Bioinformatics.

[R8] Ozaki Yoshihiko, Tanigaki Yuki, Watanabe Shuhei, Nomura Masahiro, Onishi Masaki (2022). Multiobjective Tree-Structured Parzen Estimator. Journal of Artificial Intelligence Research.

[R9] Paci Giulia, Berkemeier Francisco, Baum Buzz, Page Karen M., Mao Yanlan (2026). 3D epithelial cell topology tunes signaling range to promote precise patterning. Proceedings of the National Academy of Sciences.

[R10] Paci Giulia, Vicente-Munuera Pablo, Fernandez-Mosquera Inés, Miranda Álvaro, Lau Katherine, Zhang Qingyang, Barrientos Ricardo, Mao Yanlan (2025). Single cell resolution 3D imaging and segmentation within intact live tissues. npj Imaging.

[R11] Soelistyo CJ, Ulicna K, Lowe AR (2023). Machine learning enhanced cell tracking.. Front Bioinform.

[R12] Stringer C, Wang T, Michaelos M, Pachitariu M (2020). Cellpose: a generalist algorithm for cellular segmentation.. Nat Methods.

[R13] Tetley RJ, Staddon MF, Heller D, Hoppe A, Banerjee S, Mao Y (2019). Tissue Fluidity Promotes Epithelial Wound Healing.. Nat Phys.

[R14] Ulicna Kristina, Vallardi Giulia, Charras Guillaume, Lowe Alan R. (2021). Automated Deep Lineage Tree Analysis Using a Bayesian Single Cell Tracking Approach. Frontiers in Computer Science.

[R15] Ulman V, Maška M, Magnusson KEG, Ronneberger O, Haubold C, Harder N, Matula P, Matula P, Svoboda D, Radojevic M, Smal I, Rohr K, Jaldén J, Blau HM, Dzyubachyk O, Lelieveldt B, Xiao P, Li Y, Cho SY, Dufour AC, Olivo-Marin JC, Reyes-Aldasoro CC, Solis-Lemus JA, Bensch R, Brox T, Stegmaier J, Mikut R, Wolf S, Hamprecht FA, Esteves T, Quelhas P, Demirel Ö, Malmström L, Jug F, Tomancak P, Meijering E, Muñoz-Barrutia A, Kozubek M, Ortiz-de-Solorzano C (2017). An objective comparison of cell-tracking algorithms.. Nat Methods.

[R16] Valon L, Davidović A, Levillayer F, Villars A, Chouly M, Cerqueira-Campos F, Levayer R (2021). Robustness of epithelial sealing is an emerging property of local ERK feedback driven by cell elimination.. Dev Cell.

